# Woronin body-based sealing of septal pores

**DOI:** 10.1016/j.fgb.2017.10.006

**Published:** 2017-12

**Authors:** Gero Steinberg, Nicholas J. Harmer, Martin Schuster, Sreedhar Kilaru

**Affiliations:** a School of Biosciences, University of Exeter, Stocker Road, Exeter EX4 4QD, United Kingdom; b Donder’s Chair, University of Utrecht, Department of Biology, Padualaan 8, 3584 CH Utrecht, The Netherlands

**Keywords:** Woronin bodes, *Zymoseptoria tritici*, Septal pore, WB, Woronin body

## Abstract

In ascomycete fungi, hyphal cells are separated by perforate septa, which allow cell-to-cell communication. To protect against extensive wound-induced damage, septal pores are sealed by peroxisome-derived Woronin bodies (WBs). The mechanism underpinning WB movement is unknown, but cytoplasmic bulk flow may “flush” WBs into the pore. However, some studies suggest a controlled and active mechanism of WB movement. Indeed, in the wheat pathogen *Zymoseptoria tritici* cellular ATP prevents WBs from pore sealing in unwounded cells. Thus, cells appear to exert active control over WB closure. Here, we summarize our current understanding of WB-based pore sealing in ascomycete fungi.

## Highlights

• Upon wounding, Woronin bodies seal hyphal septa in filamentous ascomycetes.
• Little is known about the mechanism underpinning Woronin body translocation.
• Passive bulk flow of cytoplasm may move Woronin bodies into the septal pore.
• Mechanisms that involve Lah proteins are likely to support Woronin body based sealing.
• ATP is required to prevent Woronin bodies from closing pores in healthy cells.

## Video related to this article

## 1 Introduction

Most ascomycete fungi grow across substrate or tissue by invasive hyphal tip growth. The individual cells within the multicellular hyphae are separated from each other by perforate septa, with simple pores that allow exchange of cytoplasm and organelles (Abadeh and Lew, 2013). While this syncytial organization supports colony growth (Lai et al., 2012, Steinberg et al., 2017b), it also poses a serious challenge to the multi-cellular hypha. As the cytoplasmic connections facilitate passage of cell contents between cells, injury of individual cells causes extensive cytoplasmic bleeding. This, ultimately, leads to death of large parts or even the entire hypha. Fungi protect their cells from this catastrophic outcome by septal pore occlusion. This can be achieved by deposition of proteinous material or by movement of organelles into the septal pore (Markham, 1994). In both cases, a plug is formed that seals the pore and prevents cytoplasmic bleeding. The best understood mechanism of septal pore occlusion is by peroxisome-derived Woronin bodies (WB). These membrane-bound organelles Woronin bodies are found in the fungal class of Pezizomycotina, a group that includes *Neurospora crassa*, *Aspergillus fumigatus* and several plant pathogenic fungi, such as *Zymoseptoria tritici*. Whilst early studies suggested that each septum is flanked by two rings of 6 WBs (Markham, 1994), recent three-dimensional reconstruction of serial sections in *Z. tritici* have shown an irregular arrangement of 3–4 WBs, associated with the septal pore (Steinberg et al., 2017a; Video 1). WBs are positioned close to the septum by interacting with the protein Lah (see (Leonhardt et al., 2017), named after the first description of such tether LAH1/2 in *N. crassa* (Ng et al., 2009). Upon injury of a cell, WBs rapidly translocate into the septal pore, so protecting conjoined cells by stopping cytoplasmic bleeding into the wounded cell (overview in (Steinberg et al., 2017b).

Video 1Three dimensional reconstruction of a serial section image stack, showing septum-associated WBs in *Z. tritici*. The animation was generated using IMOD and ImageJ software (see Methodology). Scale bar represents 500 nm.
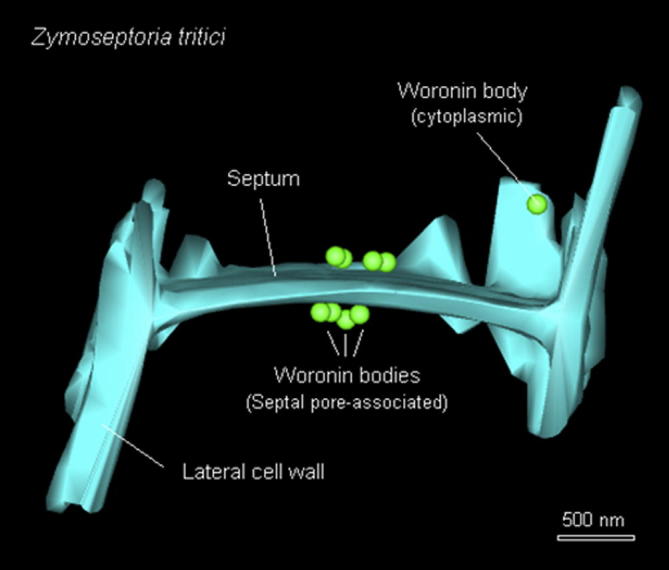

## 2 Does the cytoplasm “flush” WBs from the intact cell into the septal pore?

Fungal cells build up internal turgor pressure that drives hyphal growth (Abadeh and Lew, 2013), which drives cytoplasmic bleeding upon injury. It was widely-assumed that this wounding-induced cytoplasmic bulk flow flushes septum-associated WBs into the septal pore (Jedd and Chua, 2000, Markham and Collinge, 1987). Indeed, the short distance between WBs and the septal pore opening (100–300 nm; Han et al., 2014, Steinberg et al., 2017a), supports such a passive mechanism. Moreover, electron and light microscopy revealed that occluding WBs resist a high pressure gradient (Reichle and Alexander, 1965, Collinge and Markham, 1985, Jedd and Chua, 2000, Tenney et al., 2000, Steinberg et al., 2017a), which confirms the notion that cytoplasmic bleeding exerts forces onto the organelles. Two features of WBs allow the organelle to act as a “plug” in septal pores. Firstly, WBs are bigger that the pore. For example in *Z. tritici*, the diameter of WBs is 129 nm, whereas the septal pore opening is 41 nm (Steinberg et al., 2017a). Secondly, WBs form a crystalline core by self-assembly of the major WB protein Hex1 (Jedd and Chua, 2000; Video 2 shows a model of self-assembly of Hex1 from *N. crassa* and *Z. tritici*). This confers rigidity to the WB and thus facilitates pore plugging.

Video 2Modelling of the molecular structure and self-assembly of the putative WB protein ZtHex1. Comparison with the experimentally determined *N. crassa* Hex1 structure (Yuan et al., 2003) suggests that ZtHex1 forms similar higher-order assembles. The model was generated using MODELLER version 9.12 (see Methodology).
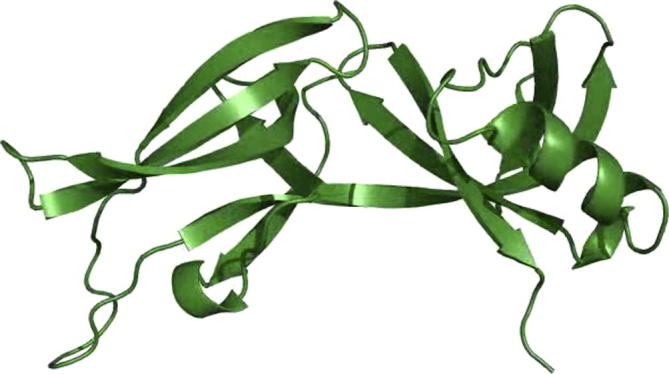

## 3 Does an active mechanism for WB-based septal pore closure exist?

WBs are generally considered to close septal pores following cell injury. However, early ultrastructural studies have shown that WBs seal 5–13% of septal pores in unwounded cells (Markham, 1994), which is thought to allow physiological heterogeneity between individual cells in fungal hyphae (Bleichrodt et al., 2012). Whilst intact *N. crassa* hypha show rapid cytoplasmic bulk flow, demonstrated by movement of organelles between hyphal cells (Abadeh and Lew, 2013), such indirect mechanism of WB translocation is unlikely to support controlled closure of WBs in unwounded cells. In fact, the identification of septum-associated protein SPA9 in *N. crassa* (Lai et al., 2012) strongly suggests a much more controlled way of moving WBs into the pore. Wounding of *N. crassa* cells results in a local WB-based closure of septa, while pores distal from the wound remain open. It was shown that these distant WBs recruit GFP-SPA9 upon injury of hyphae, suggesting a role of SPA9 in controlling WB reaction to cell rupture (Lai et al., 2012). Indeed, deletion of SPA9 in *N. crassa* resulted in impaired radial colony growth, and this growth phenotype is complemented by removal of WBs in *hex1* mutants (Lai et al., 2012). This result confirms that SPA9 is involved in preventing WBs from plugging the pore in unwounded cells. Thus, SPA9 was suggested to be inhibitor of WB activation, although the exact mechanism by which the protein exerts this control is not known (Lai et al., 2012).

## 4 Live cell imaging reveals insight into the mechanism of WB translocation

A recent study used live cell imaging of ZtHex1-eGFP fusion protein in laser-wounded cells of *Z. tritici* to provide a quantitative description of WB behavior during septal pore occlusion (Steinberg et al., 2017a). This fungus cause Septoria tritici wheat blotch disease. Despite its economic threat to agricultural industries (Fones and Gurr, 2015), we know little about its cell biology (Steinberg, 2015). The authors showed that the majority (∼85%) of the WBs close the pore from the intact cell side, which consistent with a pressure driven passive mechanism of WB translocation (Video 3). However, in 12–15% of all cases (48 laser rupture experiments), WBs closed the septum from the injured cell, thus moving against the cytoplasmic bulk flow (Steinberg et al., 2017a). This unexpected finding was also confirmed by electron microscopy. The simplest conclusion from these finding is that WB movement is based on a force-generating mechanism. It was previously considered that the cytoskeleton could participate in WB movement into the septal pore (Markham and Collinge, 1987). Steinberg et al. tested this in pharmacological experiments, but found no indication of an involvement of microtubules, F-actin or even cellular ATP in WB translocation into the pore (Steinberg et al., 2017a). Thus, WB movement towards septal pores is most unlikely to be driven by enzymes, such as molecular motors.

Video 3Live cell imaging of WBs, labelled by ZtHex1-GFP, and the septum, labelled with the plasma membrane marker mCherry-Sso1. After laser wounding, the cytoplasm leaks out of the lower cell. This creates a pressure gradient, indicated by septum bending towards the injured cell. The WB plug the septal pore on the side of the intact cell, while WBs in the ruptured cell remain associated. Time is given in seconds and milliseconds. The scale bar represents 2 µm.
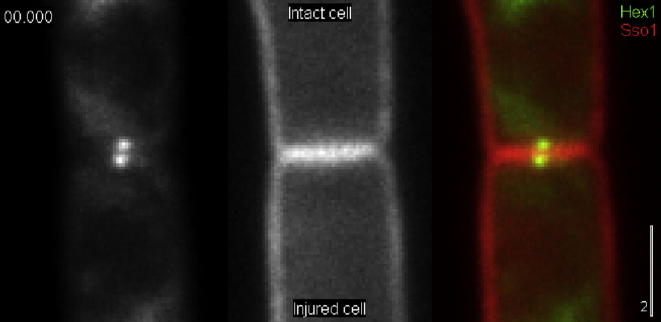

Surprisingly, depletion of ATP resulted in WB translocation into the septal pore in unwounded cells (Steinberg et al., 2017a). This suggests that ATP is required to prevent WB-based occlusion, a phenotype that is reminiscent of the SPA9 deletion mutant (Lai et al., 2012). How ATP controls WB movement remains elusive, but it has been speculated that WB-based pore occlusion may be controlled by, as yet, unknown kinases (Steinberg et al., 2017a). Alternatively, it has been speculated that ATP may bind directly to the Lah tether, thereby preventing a conformational change and contraction of the protein (Steinberg et al., 2017a). This argument was based on the presence of PVEK repeats in Lah, which are also found in the contractile muscle protein titin (Han et al., 2014, Ng et al., 2009). However, these motifs are not involved in contraction of titin (Rivas-Pardo et al., 2016). In addition, it was noted that titin binds ATP, which was thought to be indicative of a role in force generation. However, ATP-binding in titin appears to be involved in force sensing, rather than in the generation of force (Puchner et al., 2008), whereas ATP-binding in the described closure mechanism prevents WB movement. Thus, parallels between Lah and titin remain speculative and further evidence is needed to support a role of Lah proteins in ATP-dependent closure of septal pores.

## 5 Conclusions

At present, a passive, cytoplasmic bulk flow mechanism for WB movement into the septal pore cannot be excluded. Indeed, wound-induced cytoplasmic bleeding of hyphal cells causes pressure gradients that are likely to move WBs into the cytoplasmic cannels between cells. This may account for ∼85% of all septal pore closures observed in *Z. tritici* (Steinberg et al., 2017a). However, increasing evidence accumulates that an active and force-dependent mechanism may be involved in WB movement into the pore. Currently, we have very limited insight into such a mechanism. It is possible that Spa9 protein is involved, as this protein is required to keep WBs out of the septal pore in *N. crassa* (Lai et al., 2012). Studies in *Z. tritici* show that cellular ATP is required to keep the pores open, which suggests that cells detect injury by a drop in cellular ATP levels. How this change in ATP concentration induces WB movement is not understood. One possibility maybe that unknown kinase/phosphatase activity controls the pore closure. In *A. nidulans*, NIMA kinase is involved in septal pore closure, but it this kinase is not involved in WB activity (Shen et al. 2014). Another option is an ATP-dependent protein conformational change, which may involve Lah or unknown anchorage proteins. However, all these options are highly speculative and further experiments are needed to understand the mechanism of septal pore closure and the involvement of ATP.

So, why do we need to understand this mechanism better? As plant and animal pathogens, filamentous ascomycetes pose serious threats to humans (Fisher et al., 2012). Thus, WB-based occlusion of septal pores is a fungal-specific mechanism that may provide unique ways of controlling such pathogens. Indeed, recent research has begun to elucidate WB function in the human pathogen *A. fumigatus* (Beck et al., 2013, Leonhardt et al., 2017) and wheat pathogen *Z. tritici* (Steinberg et al., 2017a, Steinberg et al., 2017b). Moreover, reversible WB-based occlusion of septa may be of general importance in fungal physiology (Bleichrodt et al., 2012). Thus, elucidating the molecular details of WB-based sealing of septa may have major implications for fundamental fungal biology, but may also provide new targets for fungicide development.

## 6 Methodology

### 6.1 3D modelling of septum-assciated WBs

Liquid cultures were prepared for electron microscopy as described previously (Steinberg et al., 2017a, Steinberg et al., 2017b). Sections were investigated using a JEOL JEM 1400 transmission electron microscope operated at 120 kV and images were taken with a digital camera (ES 100 W CCD, Gatan, Abingdon, UK). The 3D-model was reconstructed from the serial micrographs using IMOD software and ETomo (http://bio3d.colorado.edu/imod/) and ImageJ (https://imagej.nih.gov/ij/).

### 6.2 Structural modelling of Hex1

Structural modeling of ZtHex1 was based on the published structure of Hex1 from *N. crassa* (PDB ID: 1KHI; Yuan et al., 2003). One complete turn of the Hex1 helix (twelve protomers) was generated from the asymmetric unit using symmetry operators. Sequence alignment was performed using CLUSTALW2, followed by minimal manual editing. The sequence identity in the modelled sequence is 77%. Comparative models were prepared using MODELLER version 9.12 (https://salilab.org/modeller/download_installation.html), using options for VTFM optimization, thorough MD optimization, and repeated optimization. The best of ten models was selected on the basis of the MODELLER energy function, and Ramachandran plot quality.
